# Good Recovery, Poor Participation? A Secondary Analysis of the Dissociation Between Global Disability and Real-Life Participation Five Years After Traumatic Brain Injury

**DOI:** 10.3390/medsci14010075

**Published:** 2026-02-09

**Authors:** Andrea Calderone, Rosaria De Luca, Tina Balletta, Lilla Bonanno, Carmela Casella, Donatella Bonaiuti, Carmela Rifici, Rocco Salvatore Calabrò

**Affiliations:** 1 IRCCS Centro Neurolesi Bonino-Pulejo, S.S. 113 Via Palermo, C.da Casazza, 98124 Messina, Italy; rosaria.deluca@irccsme.it (R.D.L.); tina.balletta@irccsme.it (T.B.); lilla.bonanno@irccsme.it (L.B.); carmela.rifici@irccsme.it (C.R.); roccos.calabro@irccsme.it (R.S.C.); 2 Stroke Unit, AOU Policlinico G. Martino, 98124 Messina, Italy; ccasella@unime.it; 3 Golgi Redaelli Institute, 20146 Milan, Italy; dbonaiuti2@yahoo.it

**Keywords:** traumatic brain injury, Glasgow Outcome Scale-Extended, participation, community reintegration, life satisfaction, mismatch profiles, vocational outcomes, long-term outcome, secondary data analysis, rehabilitation prognosis

## Abstract

**Background/Objectives**: Global disability scales such as the Glasgow Outcome Scale-Extended (GOS-E) may not fully capture real-life participation after traumatic brain injury (TBI). This secondary analysis quantified mismatch between global disability and participation 5 years after moderate-to-severe TBI and identified predictors of a “good recovery, poor participation” profile. **Methods**: We analysed the TBIMS National Database Public Use Data Set, including adults ≥ 16 years with moderate-to-severe TBI, 5-year follow-up, and valid GOS-E, PART-O, and employment data. High versus low global outcome was defined as GOS-E 7–8 versus 3–6; good versus poor participation was defined using PART-O total (≥median vs. ≤25th percentile) plus productive role engagement. Four outcome profiles were derived and compared using 2 × 2 factorial analyses and regression. **Results**: The analytic cohort included 6363 participants; among those with high GOS-E, 16.8% met criteria for poor participation. Profiles with poor participation showed lower participation and lower life satisfaction and higher mood symptoms than Group A (high GOS-E, good participation), whereas those with low GOS-E but good participation showed preserved participation with greater emotional burden. Older age, lower education, minority race/ethnicity, pre-injury unemployment or retirement, longer post-traumatic amnesia, and lower 2-year GOS-E independently predicted mismatch. Sensitivity analyses using alternative GOS-E and participation cut-offs and life-satisfaction outcomes yielded similar patterns. **Conclusions**: Five years after moderate-to-severe TBI, good global recovery does not guarantee successful reintegration, and some individuals maintain participation despite persisting disability. Routine assessment of participation and life satisfaction alongside global disability is needed to identify high-risk profiles and target vocational and psychosocial interventions.

## 1. Introduction

Traumatic brain injury (TBI) is increasingly recognized as a chronic health condition that can reshape a person’s life course long after the acute event [1,2]. Many survivors live for years with combinations of physical, cognitive, and emotional difficulties that affect their autonomy and their ability to resume pre-injury roles [1,3,4,5]. Outcomes remain highly heterogeneous even within specialized systems of acute and rehabilitation care, and long-term prognosis is difficult to anticipate at the individual level [3,4,5]. Conceptually, this chronic-disease perspective aligns with the International Classification of Functioning, Disability and Health (ICF), which distinguishes between body functions and structures, activities, participation, and contextual factors as partly independent dimensions of outcome. Global disability scales such as the Glasgow Outcome Scale-Extended (GOS-E) are widely used as primary endpoints in clinical trials and observational studies because they offer a brief, structured summary of overall functional status [6,7].

However, global outcome measures mainly describe broad levels of independence and disability and may not fully reflect how people function in their everyday environments [8]. Individuals with similar GOS-E scores can differ markedly in community integration, social relationships, and capacity to maintain productive roles [9,10]. These domains are central to long-term well-being for patients and families and frequently drive rehabilitation goals and requests for community support [11]. Previous work has suggested that favorable global outcomes do not always coincide with satisfactory participation or quality of life, indicating a more complex pattern of recovery than is conveyed by a single ordinal scale [12,13,14]. From an ICF viewpoint, GOS-E corresponds largely to the activity and global functional domain, whereas participation in social and productive roles and subjective life satisfaction represent related but distinct outcome spheres.

Participation in social roles, community activities, and productive work is a primary target of neurorehabilitation after TBI [15,16,17,18]. Instruments such as the Participation Assessment with Recombined Tools-Objective (PART-O) capture engagement in productivity, social relations, and activities outside the home, while the Satisfaction With Life Scale (SWLS) provides a global appraisal of subjective well-being [19,20,21]. These perspectives complement global disability ratings and may reveal “hidden” limitations among individuals who appear to have good recovery on the GOS-E [20,21]. The notion of mismatch describes such discordance, for example, when a person shows high GOS-E scores yet remains unemployed, socially withdrawn, or dissatisfied with life [21]. Recently, large TBI cohorts have reported discrepancies between disability and self-reported well-being [5,14], but most studies have not operationalized mismatch in a categorical way that is directly usable for clinical profiling and service planning. In particular, existing studies have typically examined continuous associations between disability, participation, and quality of life or contrasted mean scores across broad outcome strata, without deriving concrete, reproducible mismatch profiles at the level of individual patients. To our knowledge, no prior work has defined and compared multidimensional mismatch groups that simultaneously incorporate global outcome, objective participation, and productive role status several years after moderate-to-severe TBI.

The United States Traumatic Brain Injury Model Systems (TBIMS) National Database is a large, prospective, multicenter registry of individuals with TBI treated in specialized acute and inpatient rehabilitation centers [22,23]. Participants are followed at fixed time points, including 1, 2, and 5 years post-injury and at subsequent five-year intervals [22]. The TBIMS dataset includes detailed information on injury characteristics, acute care and rehabilitation, and a wide range of long-term outcomes, including GOS-E, participation measures, employment status, and life satisfaction [24,25]. This resource provides a unique opportunity to examine patterns of concordance and discordance between global disability and real-life participation several years after injury [26,27,28]. In particular, it allows the construction of categorical outcome profiles that combine global disability and participation and the characterization of these profiles in terms of sociodemographic factors, injury severity, early outcome, and psychosocial variables. Previous TBIMS-based analyses have characterized longitudinal GOS-E trajectories or participation outcomes separately [26,27,28], but this level of detail has not yet been exploited to derive categorical mismatch profiles that directly link combined patterns of global disability and participation to long-term rehabilitation needs.

The present hypothesis-driven secondary analysis used TBIMS data to quantify and characterize the mismatch between global disability, participation, and life satisfaction five years after moderate-to-severe TBI. By explicitly cross-classifying these complementary domains, the study moves beyond simply documenting associations between disability, participation, and life satisfaction to propose a pragmatic taxonomy of recovery profiles that is intended to be usable in routine follow-up and in the design of rehabilitation trials. Within an ICF-based framework, we conceptualized GOS-E as a global functional outcome measure (activities and overall independence), PART-O and productive role status as indicators of participation, and SWLS as a measure of subjective life evaluation. The primary objective was to estimate the frequency of distinct outcome profiles defined by combinations of high or low global outcome and good or poor participation, including a core group with high GOS-E but poor participation. This categorical typology yielded four clinically interpretable groups: concordant good outcome (Group A: high GOS-E, good participation), mismatch with “good recovery, poor participation” (Group B: high GOS-E, poor participation), concordant poor outcome (Group C: low GOS-E, poor participation), and mismatch with “low global outcome, good participation” (Group D: low GOS-E, good participation). Secondary objectives were to identify sociodemographic, injury-related, and rehabilitation-related predictors of mismatch profiles, with particular focus on factors associated with poor participation among individuals with high GOS-E, and to examine life satisfaction as a separate dimension of outcome. In addition to participation-based mismatch, we explored a parallel mismatch between high GOS-E and low life satisfaction, irrespective of participation level, to better understand how subjective well-being diverges from functional status. It was hypothesized that a nontrivial proportion of participants would show good global outcome yet poor participation, that this profile would be associated with selected premorbid, severity, and rehabilitation variables, and that the main conclusions would remain robust in sensitivity analyses using alternative definitions of favorable global outcome, participation, and life satisfaction. More broadly, this work aims to translate the conceptual notion of a “disability-participation paradox” into an operational framework that can support risk stratification and service planning in long-term TBI care.

## 2. Materials and Methods

### 2.1. Study Design, Data Source and Setting

This observational, multicenter study is a hypothesis-driven secondary analysis of the TBIMS National Database Public Use Data Set. The TBIMS program comprises a network of specialized centers across the United States that provide acute medical care and comprehensive inpatient rehabilitation for individuals with moderate-to-severe TBI. Eligible patients are enrolled during the index hospitalization and are followed at standardized time points, including 1, 2, and 5 years after injury and every 5 years thereafter. For the present work, we treated the 5-year follow-up assessment as a cross-sectional outcome wave and used earlier data, including 2-year GOS-E, as predictors rather than modeling full longitudinal trajectories.

The present analysis used the TBIMS National Database Public Use Data Set (DOI: 10.17605/OSF.IO/A4XZB) which was accessed/downloaded on 2 December 2025. The dataset contains de-identified longitudinal data from participating centers. No new data were collected. All variables were obtained from existing TBIMS forms, including acute care, rehabilitation, and follow-up assessments. Analyses were conducted on de-identified records provided under a formal data use agreement. The design, analysis, and reporting followed current recommendations for observational studies using routinely collected health data, including the Strengthening the Reporting of Observational Studies in Epidemiology (STROBE) and the Reporting of Studies Conducted Using Observational Routinely Collected Data (RECORD) guidelines [29,30].

### 2.2. Study Population

**Figure 1:**
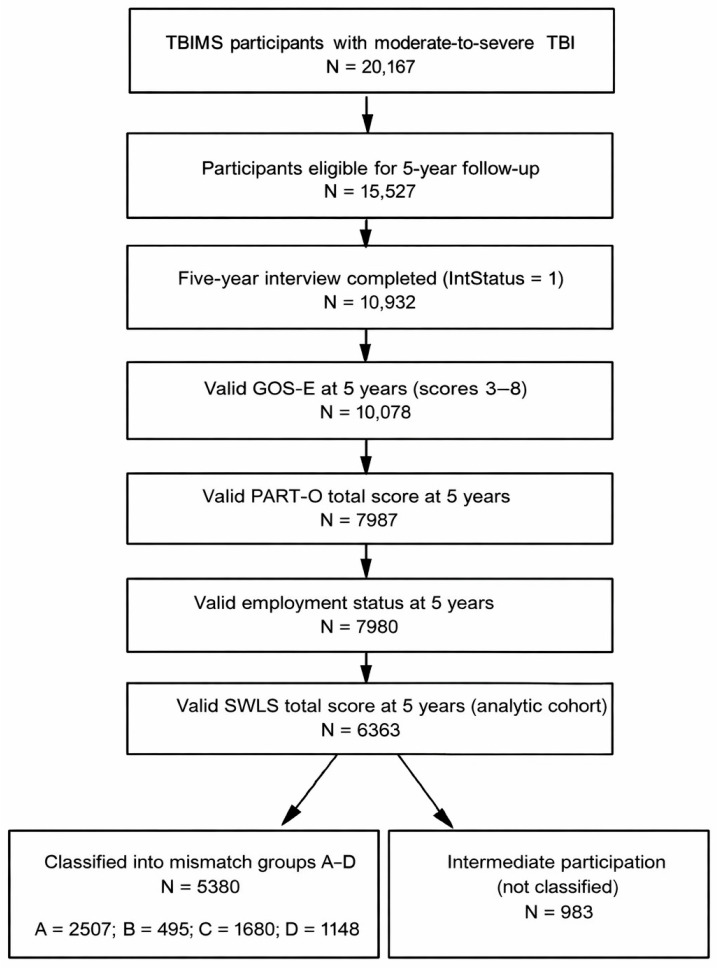
Flow of participants from the TBIMS National Database into the 5-year analytic cohort and mismatch groups. The diagram shows the sequential application of eligibility and data-quality criteria, from all 20,167 participants with moderate-to-severe TBI at baseline through 5-year follow-up, completed interview, and availability of valid GOS-E, PART-O, employment status, and SWLS scores. The final analytic cohort comprised 6363 individuals, of whom 5380 were classified into mismatch groups A–D, defined by combinations of high versus low global outcome and good versus poor participation, while 983 participants had intermediate participation and were not assigned to a mismatch group. Intermediate participation was defined as PART-O between the 25th percentile and the cohort median among participants not classified as unemployed/not working due to health reasons.

The source population comprised adults with moderate-to-severe TBI enrolled in the TBIMS National Database who received acute hospital care and comprehensive inpatient rehabilitation at participating centers (N = 20,167). Moderate-to-severe TBI was operationalized according to TBIMS criteria based on acute injury characteristics, including initial Glasgow Coma Scale (GCS) score in the moderate or severe range, presence of intracranial lesions on neuroimaging, neurosurgical intervention such as craniotomy or craniectomy, and duration of post-traumatic amnesia (PTA) consistent with at least moderate injury. For this secondary analysis, individuals were eligible if they were aged 16 years or older at the time of injury, survived to at least 5 years post injury, and completed the relevant baseline and 5-year follow-up assessments. Inclusion required valid 5-year data on GOS-E, PART-O, current employment or productive role status, and SWLS. Because participation and psychosocial measures are collected at interview, the analytic cohort is conditional on survival and retention to the 5-year assessment. TBIMS-specific missing codes and out-of-range values were handled according to the official data dictionary. Values such as 66, 88, 96, 99, 9999, and similar non-substantive codes were treated as missing for analytic purposes. Participants were excluded if they had missing, noninterpretable, or clearly implausible values for key variables, including GOS-E at 5 years, PART-O total score, employment status, SWLS score, or age at injury, or if data inconsistencies could not be resolved using the data documentation. After application of these criteria, the final analytic cohort comprised N = 6363 individuals with moderate-to-severe TBI and complete information on global outcome and participation at 5 years. A flow diagram (Figure 1) summarizes the selection of the analytic sample from the broader TBIMS dataset.

### 2.3. Outcome Measures

Global disability was assessed using the Glasgow Outcome Scale Extended (GOS-E), an ordinal scale with eight categories ranging from death to upper good recovery [6]. The scale captures overall functional status based on independence in activities of daily living, capacity for work and social participation, and presence of residual symptoms. TBIMS follow-up assessments include interviewer-rated GOS-E at 2 and 5 years post-injury. The present analysis used GOS-E at 5 years as the primary indicator of global outcome and GOS-E at 2 years as an early outcome marker. For the primary analysis, high global outcome at 5 years was defined as GOS-E scores of 7 or 8, representing lower or upper good recovery, while low global outcome was defined as GOS-E scores from 3 to 6, representing lower severe disability through upper moderate disability. Sensitivity analyses used an alternative definition of favorable outcome that classified GOS-E scores of 5 to 8 as high global outcome, consistent with prior work that treats lower moderate disability and above as favorable. Participation at 5 years was measured using the PART-O, which is administered as part of the TBIMS follow-up battery [31]. The PART-O includes domains that assess productivity, social relations, and activities outside the home, as well as a total score that reflects overall participation. Domain and total scores were calculated according to published scoring procedures. PART-O total Rasch scores range from 0 to 100, with higher scores indicating greater societal participation. For descriptive purposes, PART-O domain scores were retained on their Rasch logit scale (which can take negative values), whereas the PART-O total is reported as the 0–100 transformed Rasch score. In the present analysis, the PART-O total score served as the main indicator of participation, supplemented by information on current employment or productive role status collected at the 5-year follow-up. Employment and productive role categories included competitive employment, student status, full-time homemaker, and other roles indicating active engagement in work or study, contrasted with unemployment and not working due to health reasons.

To avoid overlapping classifications, participation categories were assigned hierarchically. Participants were first classified as having poor participation if they (i) reported being unemployed/not working due to health reasons at the 5-year follow-up or (ii) had a PART-O total score ≤ the 25th percentile of the analytic cohort. Because unemployment/not working due to health reasons contributes directly to the definition of poor participation, between-group differences in employment across mismatch groups are partly definitional (“by construction”). Among the remaining participants, good participation was defined as having a PART-O total score ≥ the cohort median. Participants who met neither criterion (i.e., PART-O between the 25th percentile and the median and not unemployed/not working due to health reasons) were classified as having intermediate participation and were not assigned to a mismatch group in the primary analyses. These thresholds were specified a priori to create clinically interpretable contrasts between good and poor participation and to preserve clear separation between participation profiles. Mismatch groups were defined by cross-classifying 5-year global outcome (high: GOS-E 7–8; low: GOS-E 3–6) with participation (good vs. poor): Group A = high GOS-E/good participation; Group B = high GOS-E/poor participation; Group C = low GOS-E/poor participation; Group D = low GOS-E/good participation. Operational definitions for participation categories and mismatch groups are summarized in Supplementary Table S1 to improve readability. Life satisfaction was assessed with the Satisfaction With Life Scale (SWLS), a five-item self-report instrument that yields a total score reflecting global cognitive judgments of life satisfaction [32]. Each item is rated on a Likert-type scale, and total scores are obtained by summing item responses. Higher scores indicate greater life satisfaction. Possible SWLS total scores range from 5 to 35 in this cohort. For the present analysis, SWLS was treated as a separate dimension of outcome rather than merged into the participation construct. Symptoms of depression at 5 years were assessed using the Patient Health Questionnaire-9 (PHQ-9), a nine-item self-report scale with total scores ranging from 0 to 27, where higher scores indicate more severe depressive symptoms. Anxiety symptoms were assessed with the Generalized Anxiety Disorder-7 (GAD-7), a seven-item self-report scale with total scores from 0 to 21, where higher scores indicate more severe anxiety. Clinically significant depressive and anxiety symptoms were defined as PHQ-9 and GAD-7 total scores ≥ 10, respectively, consistent with established thresholds. Rehospitalization in the year preceding the 5-year follow-up was coded as a binary indicator (any vs. no hospital admission). Social context at 5 years was described using living situation (spouse or partner, alone, other family, someone else) and marital status (single, married, divorced, separated, widowed, other), as reported in the TBIMS follow-up forms. Low life satisfaction was defined as an SWLS total score ≤ 14, consistent with commonly used cutoffs that indicate dissatisfaction in TBI and general population samples. This operationalization allowed examination of the mismatch between global outcome and life satisfaction among participants with high GOS-E, including those with acceptable participation. Four mutually exclusive mismatch groups were derived using the primary definitions of high versus low global outcome and good versus poor participation at 5 years. Group A included participants with high GOS-E and good participation and represented a concordant good outcome profile. Group B included participants with high GOS-E but poor participation and represented the core “good recovery, poor participation” mismatch profile. Group C included participants with low GOS-E and poor participation and reflected a concordant poor outcome profile. Group D included participants with low GOS-E and good participation and was interpreted as a profile in which individuals reported relatively favorable participation despite persisting global disability, possibly due to compensatory effects of environmental, family, or personal factors. For sensitivity analyses, additional cross-classifications were created within the high GOS-E stratum to distinguish participants with high versus low SWLS, allowing exploration of mismatch between global outcome and life satisfaction among those with favorable global disability scores. Several other variables were included as covariates or descriptive characteristics. Demographic variables comprised age at injury, sex, race or ethnicity, and pre-injury education. Pre-injury characteristics included employment status, substance use, and other available indicators of premorbid social and health status. Injury-related variables included mechanism of injury, initial GCS score, presence of intracranial lesions, duration of post-traumatic amnesia, intracranial pressure monitoring, and neurosurgical procedures such as craniotomy or craniectomy. Rehabilitation-related variables included time from injury to rehabilitation admission, length of stay in acute care and inpatient rehabilitation, discharge destination, and payor source. Where available, early functional trajectory classes derived from longitudinal functional measures in the first two years were used as additional predictors that summarized early recovery patterns. Several variables were recoded to improve clinical interpretability, for example, categorizing post-traumatic amnesia into standard duration bands and expressing lengths of stay in units of ten days.

### 2.4. Statistical Analysis

All statistical analyses were conducted using R, version 4.2.1 (R Foundation for Statistical Computing, Vienna, Austria), with two-sided tests and a significance threshold of *p* < 0.05 [33]. Data preparation involved merging baseline (TBIMS Form 1) and follow-up (TBIMS Form 2) records using the unique participant identifier, restricting the dataset to participants with a completed 5-year interview, and applying the inclusion and exclusion criteria described above. Coding and valid ranges of key variables were verified against the TBIMS data dictionary and codebooks. Values that represent non-substantive responses in the TBIMS documentation, including variable-specific codes such as 66, 88, 99, 777, 888, 999, 8888, and 9999, were treated as missing as appropriate. Descriptive checks of distributions, outliers, and logical consistency were performed prior to analysis. Because the analytic cohort was defined by complete 5-year outcome data, missingness in the primary models primarily reflects item-level missingness in covariates among otherwise eligible 5-year respondents. Primary regression models therefore used complete-case data for covariates included in each model, and estimates should be interpreted as conditional on complete covariate information.

Mismatch groups were constructed by cross-classifying global outcome and participation at 5 years using the operational definitions in Section 2.3, which are summarized in Supplementary Table S1. Descriptive statistics were calculated for the overall analytic cohort and for each mismatch group. Continuous variables were summarized using means and standard deviations when approximately symmetric and with medians and interquartile ranges when distributions were clearly skewed. Categorical variables were summarized using counts and percentages. Group differences were examined within a factorial 2 × 2 framework because groups A–D represent the full cross-classification of global outcome (high vs. low GOS-E) and participation (good vs. poor). Continuous variables were evaluated using two-way analysis of variance models that included the interaction term. Tukey-adjusted post hoc comparisons were used when follow-up pairwise contrasts were needed, with particular attention to the contrast between Group B and Group A to isolate factors associated with poor participation among individuals with high GOS-E. Categorical variables were compared using chi-square tests. For graphical comparison of psychosocial outcomes across mismatch groups, selected measures (PART-O total, SWLS total, PHQ-9, GAD-7, and a binary indicator of no rehospitalization) were standardized to z-scores using the mean and standard deviation of the analytic cohort. PHQ-9 and GAD-7 were multiplied by −1 so that higher standardized values consistently reflected more favorable outcomes.

To identify predictors of mismatch group membership, multinomial logistic regression models were fitted with mismatch group as the dependent variable and Group A as the reference category. These models were restricted to participants assigned to Groups A–D, with the intermediate participation category excluded from mismatch-group models in the primary analyses. Candidate predictors included demographic factors, pre-injury characteristics, injury severity markers, rehabilitation-related variables, and early outcome measures such as 2-year GOS-E. Results are reported as adjusted odds ratios with 95% confidence intervals. A secondary binary logistic regression focused on the high GOS-E stratum and contrasted Group B versus Group A using the same predictor set subject to parsimony constraints. Sensitivity analyses examined alternative definitions of favorable global outcome and participation thresholds. Across all analyses, interpretation emphasized the direction and magnitude of differences and associations rather than *p*-values alone.

### 2.5. Ethics

The study used only deidentified data from the TBIMS National Database Public Use Data Set. The original TBIMS protocols at each participating center were approved by institutional review boards or ethics committees, and informed consent was obtained from participants or their legally authorized representatives in accordance with local regulations. The Public Use Data Set is fully de-identified prior to release by the TBIMS National Data and Statistical Center and does not contain direct identifiers or linkage codes that would permit re-identification of individual participants. Because the study relied exclusively on fully anonymized data obtained from an open-access online database, and no identifiable or sensitive personal information was processed, local ethics committee approval was not required, although all relevant ethical and data-privacy principles were respected. All analyses were conducted in accordance with the principles of the Declaration of Helsinki and applicable regulations governing the secondary use of anonymized health data [34].

## 3. Results

The initial TBIMS cohort included 20,167 participants with moderate-to-severe TBI, of whom 15,527 were eligible for 5-year follow-up and 10,932 completed the 5-year interview. After restricting to individuals with valid GOS-E scores between 3 and 8, PART-O Rasch scores, employment status, and SWLS totals at 5 years, the analytic cohort comprised 6363 participants. The analytic sample had a mean age at injury of 38.6 ± 17.2 years and was predominantly male and White (72.1% male; 71.4% White). Motor vehicle or transport-related events were the most common cause of injury, followed by falls, and 4353 participants (68.6%) were employed before injury. Acute and rehabilitation lengths of stay were substantial, and almost one quarter had undergone craniotomy or craniectomy. These characteristics are summarized in Table 1.

**Figure 2:**
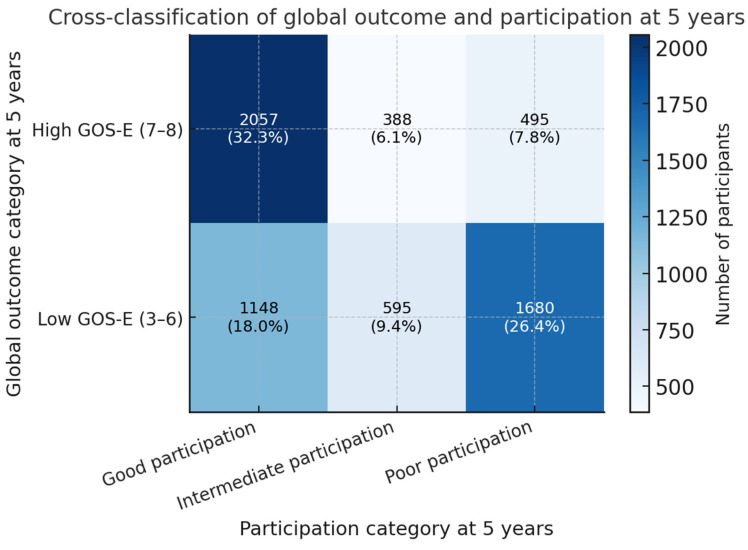
Cross-classification of global outcome and participation at 5 years after moderate-to-severe TBI. The heatmap shows the distribution of participants in the analytic cohort (N = 6363) across categories of global outcome, defined as high GOS-E (scores 7–8) versus low GOS-E (scores 3–6), and participation at 5 years, defined as good, intermediate, or poor based on PART-O total scores and current productive role. Cell labels report the number of participants and the percentage of the analytic cohort in each combination.

The joint distribution of global outcome and participation at 5 years showed marked heterogeneity. Among the 6363 participants, 2940 had high GOS-E scores of 7 or 8, and 3423 had scores between 3 and 6. Within the high GOS-E group, 2057 individuals, corresponding to 70.0% of this stratum and 32.3% of the full cohort, met criteria for good participation and formed Group A. A further 495 participants, representing 16.8% of those with high GOS-E and 7.8% of the overall cohort, met criteria for poor participation and constituted the core “good recovery, poor participation” Group B. The remaining 388 individuals with high GOS-E had intermediate participation and were not classified into the four mismatch groups. Within the low GOS-E stratum, 1680 participants, or 49.1% of that stratum and 26.4% of the full cohort, had poor participation and were assigned to Group C, whereas 1148 participants, corresponding to 33.5% of those with low GOS-E and 18.0% of the cohort, achieved good participation and were assigned to Group D. A further 595 individuals with low GOS-E had intermediate participation. Overall, 5380 participants were classified into the four mismatch groups, and 983 had intermediate participation. Participants with intermediate participation were not included in the primary mismatch-group models, which were restricted to the four clearly separated profiles (Groups A–D). Taken together, 34.2% of the full cohort (Groups B and C combined) had poor participation, whereas 50.3% (Groups A and D combined) achieved good participation despite heterogeneity in global disability. The cross-classification of GOS-E categories with participation categories is illustrated in Figure 2.

Baseline demographic and injury characteristics differed across the four mismatch groups in clinically meaningful ways (Table 2).

Participants in Group A were the youngest on average, with a mean age at injury of 34.6 years, whereas those in Group B and Group C were older, with mean ages of 43.8 and 40.8 years, and Group D showed an intermediate profile. Two-way analysis indicated that age differences were primarily associated with participation status and varied by global outcome stratum, with a significant global outcome-by-participation interaction. Years of education were highest in Group A and lowest in Group C. Factorial models showed independent main effects of both global outcome and participation on education and no evidence of interaction. Markers of injury severity and acute care utilization aligned more closely with global outcome than participation. Initial GCS was modestly higher in the high GOS-E stratum, whereas post-traumatic amnesia and acute and rehabilitation lengths of stay were shortest in Group A and longest in Group C. Two-way models confirmed strong main effects of global outcome for post-traumatic amnesia and length-of-stay outcomes, with smaller additional participation effects for length of stay.

The distribution of pre-injury sociodemographic characteristics also differed by group. Participants in Group A and Group D were most likely to have been competitively or specially employed before injury, whereas Group B and Group C contained higher proportions of individuals who were retired or unemployed. Nearly one-third of Group B was retired at the time of injury. Among these participants, 128 of 139 (92.1%) were also coded as retired at 5 years, and only 6 (4.3%) were coded as unemployed, indicating that their assignment to poor participation was driven mainly by low PART-O scores rather than unemployment status. Non-White race or ethnicity was more frequent in Groups B and C than in Groups A and D. Falls and violence-related injuries were more common in Groups B and C, whereas motor vehicle or transport injuries predominated in Groups A and D. Craniotomy or craniectomy was most frequent in Group C, consistent with longer post-traumatic amnesia and longer lengths of stay. Pre-injury illicit drug use was also most prevalent in Group C and least common in Group A. Five-year outcomes reflected the defining mismatch patterns as well as additional differences beyond the construction of the groups (Table 3).

By design, Groups A and B had similarly high global outcome scores at 5 years, with mean GOS-E scores of 7.7 and 7.6, whereas Groups C and D had lower mean GOS-E scores of 4.7 and 5.5 (Table 3). Two-year GOS-E scores already differed across groups, with Group A showing the highest early global outcome and Group C the lowest, and two-way analysis supporting independent main effects of both global outcome and participation with a small interaction. Participation outcomes showed larger contrasts between groups that shared similar GOS-E levels. The PART-O total score at 5 years was highest in Group A and nearly as high in Group D despite lower GOS-E scores in Group D, whereas Group B and Group C had markedly lower PART-O totals. Factorial models indicated independent associations of both global outcome and participation with PART-O measures and interactions for the total score and most domains (Supplementary Table S1), while differences in the Social Relations domain were comparatively modest. The employment distribution mirrored these patterns. Because unemployment contributed to the operational definition of poor participation, between-group differences in employment are partly definitional. In Group A, more than four-fifths of participants were competitively employed at 5 years, whereas only 4.6% of Group B and 3.3% of Group C achieved competitive employment. Group D showed an intermediate profile, with almost half of participants competitively employed. Unemployment at 5 years was absent in Groups A and D by definition but affected approximately half of Group B and more than one-third of Group C.

**Figure 3:**
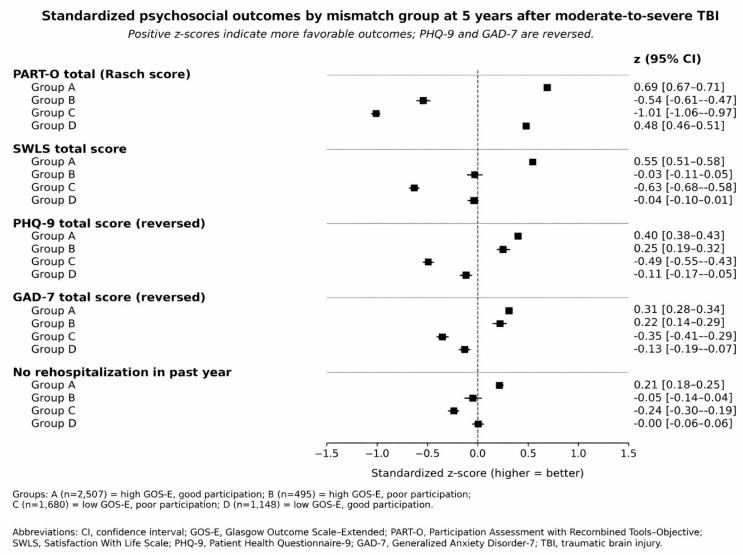
Standardized psychosocial outcomes by mismatch group at 5 years after moderate-to-severe TBI. The forest plot shows group-specific z-scores and 95% confidence intervals for participation (PART-O total Rasch score), life satisfaction (SWLS total), depressive symptoms (PHQ-9, reversed), anxiety symptoms (GAD-7, reversed), and absence of rehospitalization in the previous year. Scores are standardized to the mean and standard deviation of the analytic cohort, with zero indicating the cohort average and positive values indicating more favorable outcomes. PHQ-9 and GAD-7 were multiplied by −1 so that higher standardized values reflect fewer symptoms.

Patterns of subjective well-being, mood, anxiety, rehospitalization, and social context further differentiated the mismatch groups (Table 3). SWLS scores were highest in Group A and lowest in Group C. Group B and Group D had intermediate SWLS means around 22, yet one-fifth of participants in each group met criteria for low life satisfaction compared with 5.3% in Group A. Depressive symptoms showed a gradient across groups. Mean PHQ-9 scores were lowest in Group A and highest in Group C, with Group B and Group D again intermediate. Clinically significant depressive symptoms ranged from 5.6% in Group A to 10.4% in Group B, 22.6% in Group D, and 35.1% in Group C. Anxiety symptoms measured by GAD-7 followed a comparable pattern. Two-way analysis supported independent main effects of both global outcome and participation on SWLS, PHQ-9, and GAD-7, with evidence of an interaction for PHQ-9 but not for SWLS or GAD-7. Rehospitalization in the year before the 5-year follow-up was least frequent in Group A and most frequent in Group C, with Groups B and D showing intermediate proportions. Figure 3 presents these contrasts in standardized form and highlights the broad psychosocial disadvantage of Groups B and C compared with Group A, as well as the mixed profile of Group D.

Social context variables suggested that relationship status and living arrangements may partly distinguish the mismatch profiles. Participants in Group A and Group D were more often living with a spouse or partner, whereas Group B and Group C contained higher proportions of people living alone or with other family members. Widowed and divorced marital status categories were more frequent in Group B and Group C, while marriage was most common in Group A and Group D. These differences may reflect both pre-injury social resources and the downstream impact of injury and disability on family structures.

Multivariable regression models quantified independent predictors of mismatch group membership relative to Group A (Table 4). In the multinomial model restricted to complete cases (n = 2257), older age was associated with greater odds of belonging to each of the three other groups. Each 10-year increase in age at injury was associated with higher odds of Group B membership (adjusted OR 1.43, 95% CI 1.26–1.62), Group C membership (adjusted OR 1.34, 95% CI 1.21–1.48), and Group D membership (adjusted OR 1.16, 95% CI 1.05–1.28) compared with Group A. Non-White race or ethnicity predicted membership in Groups B and C, and lower education was associated with higher odds of poor participation profiles. Pre-injury employment patterns showed strong associations. Participants who were retired or unemployed before injury were substantially more likely to belong to Group B or Group C than to Group A, with adjusted odds ratios exceeding five for pre-injury retirement in Group B and nearly three for pre-injury unemployment. Pre-injury student status was also associated with higher odds of belonging to Group B. Male sex and pre-injury homemaker status were associated with lower odds of belonging to Group D compared with Group A. Pre-injury illicit drug use was associated with higher odds of membership in Groups B and C and showed a weaker, non-significant trend for Group D.

**Figure 4:**
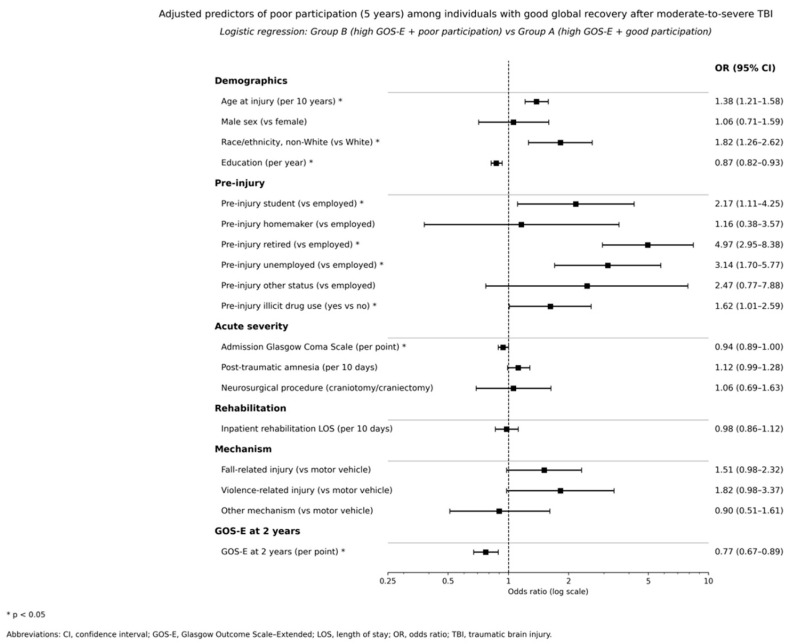
Adjusted predictors of poor participation among individuals with good global recovery at 5 years after moderate-to-severe TBI. The forest plot displays adjusted odds ratios (aORs) with 95% confidence intervals from the multivariable logistic regression model comparing Group B (high GOS-E and poor participation) with Group A (high GOS-E and good participation). Predictors include age at injury (per 10 years), sex, race or ethnicity, education (years), pre-injury employment category, pre-injury illicit drug use, admission GCS, post-traumatic amnesia duration (per 10 days), inpatient rehabilitation length of stay (per 10 days), craniotomy or craniectomy, injury mechanism, and GOS-E at 2 years (per 1-point increase). Values greater than 1 indicate higher odds of belonging to Group B relative to Group A, whereas values lower than 1 indicate lower odds of poor participation among individuals with favorable global outcomes.

Injury severity and early outcome variables retained independent associations after adjustment. Longer post-traumatic amnesia increased the odds of belonging to Group C and Group D compared with Group A, whereas the corresponding association for Group B did not reach statistical significance. Inpatient rehabilitation length of stay did not show an independent association once other factors were included. Neurosurgical procedures were associated with higher odds of belonging to Group C, consistent with greater injury complexity in the concordant low outcome profile. Admission GCS showed a modest protective association in the binary model contrasting Group B with Group A within the high GOS-E stratum (adjusted OR 0.94 per point, 95% CI 0.89–1.00). The mechanism of injury displayed only weak and mostly nonsignificant associations after adjustment. The strongest predictor across all multinomial contrasts was GOS-E at 2 years. Higher early global outcome scores were associated with substantially lower odds of later mismatch, with adjusted odds ratios per one-point increase ranging from 0.70 for Group B to 0.33 for Group C and 0.43 for Group D compared with Group A. In the binary model focusing on poor participation among individuals with good global recovery, each one-point increase in 2-year GOS-E was associated with lower odds of Group B membership (adjusted OR 0.77, 95% CI 0.67–0.89). These findings are detailed in Table 4 and visually summarized for the contrast between Group B and Group A in Figure 4.

Sensitivity analyses that used an alternative definition of favorable global outcome starting at GOS-E scores of 5 and alternative cutoffs for good and poor participation based on PART-O distributions produced similar patterns. The proportion of participants classified as having good recovery with poor participation changed modestly with these definitions, yet a distinct subgroup with high global outcome and low participation remained evident. The direction and relative magnitude of associations between key predictors and mismatch profiles were stable across model specifications, and analyses focusing on low life satisfaction rather than poor participation identified a partly overlapping subgroup with discrepant outcomes on global disability versus subjective well-being.

## 4. Discussion

This secondary analysis shows that global disability and real-life participation often diverge five years after moderate-to-severe TBI. A sizeable minority of participants who reached good recovery on the GOS-E still had poor participation, while another subgroup achieved relatively good participation despite persisting disability. The four mismatch profiles captured these patterns in a simple and clinically interpretable way. The findings support the view that global outcome scales describe an important but incomplete slice of long-term recovery, and that participation and life satisfaction require explicit assessment in their own right. Taken together, approximately one-third of the cohort had poor participation, and about one-half achieved good participation, underscoring that these discrepant profiles are common rather than exceptional. These findings complement prior work on discrepancies between disability ratings and subjective well-being after TBI [14] by extending the focus to objectively defined participation profiles in a large, multicenter cohort. Unlike previous studies that have focused primarily on either self-reported well-being or isolated participation indices [5,14], the present analysis defines four a priori profiles that jointly capture global disability, objective participation, and productive role status at 5 years within a unified ICF-based framework.

The core focus of the study was the “good recovery, poor participation” profile. Around one in six individuals with high GOS-E scores fell into Group B, reflecting low PART-O scores, very low rates of competitive employment, and frequent unemployment attributed to health (a definitional component of the poor-participation classification). Most individuals in Group B who were retired before injury remained retired at 5 years, and poor participation in this subgroup primarily reflected low participation scores rather than an expectation of return to work. In absolute terms, this group represented 7.8% of the full analytic cohort, reinforcing that “good recovery, poor participation” constitutes a relatively frequent chronic outcome profile in specialized TBI services rather than an outlier pattern. To our knowledge, this is the first TBIMS-based study to quantify the prevalence and correlates of this “good recovery, poor participation” phenotype using explicit, reproducible criteria that combine GOS-E, PART-O, and productive role status at a fixed long-term time point. These participants had early global outcomes similar to Group A yet experienced marked constraints in social and productive roles, consistent with prior work showing that return to work and sustained employment after TBI are influenced by multiple clinical and contextual factors [35,36]. This pattern resonates with reports of discrepancy between disability and reported well-being after TBI [14], while emphasizing that substantial limitations in objective participation can coexist with apparently favorable global disability scores. Life satisfaction and mood were also worse than in Group A, although not as poor as in the concordant low outcome group. This pattern suggests that returning to a broadly independent level of functioning does not guarantee restoration of complex social roles. Structural barriers, labor market dynamics, premorbid vulnerabilities, and persistent cognitive or behavioral changes that are not fully captured by the GOS-E probably contribute to this disconnect, highlighting the potential role of targeted cognitive and vocational rehabilitation and structured return-to-work interventions in mitigating long-term participation failure [37,38,39].

The group with low GOS-E but good participation provides a complementary perspective. Participants in Group D reported PART-O scores and employment rates close to those of Group A despite substantially lower global outcome scores, echoing evidence that some individuals with significant residual deficits can nonetheless achieve satisfactory levels of community integration and societal participation [40,41,42]. Many remained in roles that demand social and cognitive effort, such as employment, study, or active homemaking. At the same time, mood symptoms, anxiety, and rehospitalization were more frequent than in Group A and resembled Group C more closely, in line with studies showing that high participation does not necessarily imply low psychological burden [43]. This pattern suggests that environmental supports, family resources, and personal coping strategies can enable individuals with ongoing disability to sustain participation, yet often at emotional cost. The existence of this group underlines the risk of underestimating psychological burden and service needs when outcome assessment relies only on participation indicators. By delineating this profile alongside the “good recovery, poor participation” group, the present study adds nuance to the disability paradox literature by showing that favorable participation does not uniformly coincide with low psychological morbidity or health-care utilization. From a service-planning perspective, Group D may represent a form of “positive deviance,” in which high engagement in work and social roles is maintained at the cost of increased psychological and medical burden, suggesting a need for ongoing monitoring and supportive interventions even when participation appears successful.

Psychosocial profiles across the four groups highlight the layered nature of post-TBI adjustment. Group C, defined by low GOS-E and poor participation, carried the highest burden across almost all adverse outcomes, including depressive and anxiety symptoms, low life satisfaction, and rehospitalization. Group B occupied an intermediate position between Groups A and C, with better mood and life satisfaction than Group C yet clearly worse than Group A. Group D showed a mixed pattern, with participation and life satisfaction close to Group A but mood and rehospitalization closer to Group C. These gradients, summarized in the standardized outcome plot, make clear that mismatch is not a rare statistical curiosity. Instead, it reflects distinct clinical phenotypes that require different forms of long-term support, consistent with prior work linking post-traumatic amnesia, leisure and community activities, and broader contextual factors to heterogeneous patterns of long-term adjustment [44,45,46]. Taken together, these gradients across domains reinforce the emerging view of TBI as a chronic health condition with enduring psychosocial sequelae rather than a time-limited event [1,2].

Predictor analyses identify several factors that increase the likelihood of poor participation among individuals with good global recovery. Older age, lower education, and non-White race or ethnicity were associated with higher odds of belonging to Group B rather than Group A, which is consistent with evidence that social disadvantage and minority status are linked to participation barriers after TBI [47,48]. Pre-injury retirement and unemployment showed particularly strong associations with Group B and Group C, suggesting that vocational vulnerability before injury may amplify the risk of long-term participation failure even when functional recovery appears favorable [35,40]. Pre-injury illicit drug use also increased the odds of poor participation profiles. Injury severity indices had smaller and less consistent effects in the fully adjusted models, although longer post-traumatic amnesia was associated with the concordant low outcome profile and with the good-participation, low-GOS-E profile. The most consistent predictor across multinomial contrasts was early global outcome at 2 years. Adjusted odds ratios per one-point increase in 2-year GOS-E ranged from 0.70 to 0.33 across the multinomial comparisons, and 2-year GOS-E remained protective in the binary model contrasting Group B with Group A among individuals with high 5-year GOS-E (Table 4). These results underscore that early functional recovery is prognostically informative but not sufficient and that participation outcomes several years after injury are shaped by a combination of early severity, premorbid social position, and chronic-phase contextual barriers.

Compared with earlier TBIMS and CENTER-TBI analyses that modelled single outcomes such as GOS-E or community integration [5,14,40], our multinomial approach underscores that these factors shape the likelihood of occupying specific multidimensional profiles rather than merely shifting average scores on individual scales. Identifying such profiles early may allow clinicians to prioritize intensive vocational and community-based rehabilitation for individuals most at risk of “good recovery, poor participation.” Nevertheless, the observational design precludes causal inference, and these predictors should be interpreted as markers of increased risk rather than as directly modifiable determinants of mismatch.

The results have practical implications for outcome assessment and service planning. Exclusive reliance on GOS-E or similar global scales is likely to underestimate long-term needs in roughly one quarter of survivors who appear to do well on global disability but remain disconnected from work and community roles. Routine inclusion of participation measures such as the PART-O and brief life satisfaction scales in follow-up protocols would provide a more accurate picture of recovery trajectories, in line with proposals to treat participation as a core rehabilitation outcome in its own right [49]. This approach is consistent with the ICF, which conceptualizes participation as a distinct but complementary dimension of outcome alongside impairments and activity limitations. These data also inform trial design. Interventions aimed at improving participation or quality of life should consider stratifying or targeting participants based on combined profiles of global outcome and participation rather than on global disability alone. Group B in particular represents a plausible target for vocational and psychosocial interventions that build on preserved independence but address participation barriers directly [37,38,39,49]. In practice, simple cross-classification of GOS-E and PART-O, as implemented in the present study, could be used to operationalize such profiles at the point of care and to stratify participants in future intervention trials.

### Methodological Strengths and Limitations in Context

The study draws strength from the size and richness of the TBIMS cohort. The analytic sample included more than six thousand individuals with standardized assessments at 5 years across multiple domains, which allowed robust estimation of mismatch frequencies and exploration of predictors with adequate power. Prospective enrolment during acute care and structured follow up reduce recall bias and support temporal interpretation of associations between early factors and later profiles. Use of validated instruments such as the GOS-E, PART-O, SWLS, PHQ 9, and GAD 7 increases confidence in the reliability and comparability of the measurements. The transparent operationalisation of mismatch groups, based on widely used cut points and the empirical distribution of participation scores, facilitates clinical interpretation and replication in other datasets. A further strength is that the mismatch profiles and their thresholds were specified a priori from an ICF perspective and are straightforward to implement in clinical registries or follow-up clinics, enhancing the translational relevance of the proposed taxonomy. Moreover, the study design, analysis, and reporting adhered to current recommendations for observational research using routinely collected data (STROBE and RECORD), which strengthens transparency and reproducibility.

Several limitations need consideration when interpreting these findings. The analysis focused on survivors who completed the 5-year interview and had valid data on key outcomes. Individuals with the most severe impairments, unstable living situations, or loss to follow up are likely underrepresented. The observed frequencies of mismatch may therefore underestimate the true burden in the broader population of survivors. Although the TBIMS cohort has been shown to be broadly, but not fully, representative of hospitalized TBI in the United States [24,25,28], residual selection bias is likely and should be considered when extrapolating these frequencies to other settings. In addition, adjusted regression models were restricted to participants with complete covariate data, which reduced the analytic sample and may introduce further selection bias if covariate missingness is not completely at random. The construction of mismatch groups relied on cutoffs for GOS-E and PART-O that, although grounded in prior work and supported by sensitivity analyses, remain somewhat arbitrary. Different thresholds would shift some individuals between categories, particularly those near the median of participation. The choice to exclude participants with intermediate participation from the four groups also reduced sample size for some analyses, although this approach produced more distinct and conceptually clear profiles.

Participants with intermediate participation (i.e., PART-O between the 25th percentile and the median and not unemployed/not working due to health reasons) were not assigned to mismatch groups and were therefore excluded from mismatch-group regression models; accordingly, predictor estimates pertain to the four-profile classification (Groups A–D) and may not generalize to this intermediate band.

Measurement constraints are another source of uncertainty. Participation and life satisfaction were captured at a single 5-year time point rather than longitudinally, which prevents analysis of within-person changes and the potential oscillation between profiles over time. Some domains relevant to participation, such as informal caregiving, community involvement, and cognitive endurance in complex roles, are only partially captured by the PART-O. Employment status was coded without detailed information on job demands, job quality, or workplace accommodations, and misclassification between “unemployed” for health reasons and other inactive categories cannot be fully excluded. Mood and anxiety measures were self-reported and may be influenced by insight, response style, and cultural factors. Furthermore, productivity-related participation scores may be lower among individuals who were retired before injury, and these cases should be interpreted as low participation rather than as an expectation of return to employment.

Residual confounding is likely despite adjustment for a broad range of covariates. In addition, multivariable models were restricted to participants with complete data on all predictors, which may have introduced further selection bias and limits the generalizability of the adjusted estimates. Detailed information on social determinants such as income, neighborhood characteristics, family support, and access to community services was limited. These factors probably play a major role in shaping participation and may explain part of the associations attributed to race, education, or preinjury employment. The TBIMS cohort draws from specialized centres in the United States and reflects the organization of trauma and rehabilitation services in that context. Generalizability to health systems with different funding models, social protection schemes, and community resources may be restricted. The analyses of life satisfaction mismatch were constrained by missing data and should be interpreted as exploratory, even though they broadly aligned with the participation-based profiles.

Despite these limitations, the study offers a detailed and internally coherent picture of how global outcome, participation, and subjective well-being intersect several years after moderate-to-severe TBI. The combination of descriptive profiles, cross-sectional comparisons, and multivariable models provides converging evidence that mismatch is common, predictable to some extent from early characteristics, and clinically meaningful. The large sample and rigorous outcome measurement mitigate some concerns about selection and measurement bias, while the sensitivity analyses suggest that the main conclusions are robust to alternative definitions of favorable outcome and participation particularly for key predictors such as age, early global outcome, and pre-injury employment status. In this sense, the study extends prior descriptive reports of discordant disability, participation, and well-being after TBI by offering a concrete, operational framework for stratifying long-term recovery phenotypes that can inform both clinical follow-up and the targeting of rehabilitation resources.

## 5. Conclusions

Long-term outcomes after moderate-to-severe TBI cannot be understood through global disability scales alone. This secondary analysis of the TBIMS National Database shows that a substantial minority of survivors achieve good recovery on the GOS-E yet remain poorly integrated in work and social roles, while another subgroup participates actively despite ongoing disability. These mismatch patterns are associated with age, education, race or ethnicity, pre-injury employment, early severity, and early global outcome. Participation, life satisfaction, mood, and rehospitalization add important information beyond global disability and identify distinct clinical phenotypes that are likely to have different service needs. Collectively, these observations extend emerging evidence that TBI behaves as a chronic condition with long-term consequences for social, vocational, and emotional functioning.

Routine assessment of participation and life satisfaction in long-term follow-up would improve prognostic counseling and help clinicians recognize survivors who require continued vocational and psychosocial support despite apparently favorable global outcomes. Rehabilitation programs and health systems could consider designing interventions that specifically target the “good recovery, poor participation” profile while maintaining support for individuals who sustain participation at the cost of higher emotional burden. Implementation studies will be required to determine how such stratified, profile-based interventions can be embedded effectively into routine follow-up pathways across diverse health systems. Future work could extend this approach to longer follow-up intervals, incorporate richer measures of environmental context, and test whether tailored interventions can modify mismatch trajectories. The present findings underline the need for a broader, participation-centred view of recovery after TBI that aligns outcome measurement more closely with what matters to survivors and their families and with contemporary multidimensional frameworks of disability and participation.

## Figures and Tables

**Table 1:** Sample selection and baseline characteristics of the analytic cohort at 5 years after TBI. Panel A. Selection of the analytic cohort. Panel B. Baseline demographic, injury, and rehabilitation characteristics of the analytic cohort (N = 6363).

Cohort Definition	N	% of Preceding Stage	% of Initial TBIMS Cohort
All TBIMS participants with moderate-to-severe TBI	20,167	–	100.0
Eligible for 5-year follow-up (FollowUpPeriod = 5)	15,527	77.0	77.0
Interview completed at 5 years (IntStatus = 1)	10,932	70.4	54.2
Valid 5-year GOS-E 3–8	10,078	92.2	50.0
Valid PART-O Rasch score at 5 years	7987	79.3	39.6
Valid employment status at 5 years (EMPLOYMENTF 2–12)	7980	99.9	39.6
Valid SWLS total score 5–35 at 5 years	6363	79.7	31.6
**Characteristic**		**Analytic cohort (N = 6363)**	
Age at injury, years		38.6 ± 17.2	
Male sex, n (%)		4588 (72.1%)	
Female sex, n (%)		1772 (27.9%)	
White race, n (%)		4542 (71.4%)	
Non-White race, n (%)		1818 (28.6%)	
Education, years		12.9 ± 2.7	
Mechanism: Motor vehicle/transport, n (%)		3607 (56.7%)	
Mechanism: Fall, n (%)		1448 (22.8%)	
Mechanism: Violence, n (%)		570 (9.0%)	
Mechanism: Other, n (%)		734 (11.5%)	
Pre-injury employed, n (%)		4353 (68.6%)	
Pre-injury student, n (%)		494 (7.8%)	
Pre-injury homemaker, n (%)		129 (2.0%)	
Pre-injury unemployed, n (%)		654 (10.3%)	
Pre-injury retired, n (%)		669 (10.5%)	
Pre-injury other employment status, n (%)		51 (0.8%)	
Pre-injury illicit drug use, n (%)		1326 (20.8%)	
Initial GCS total		11.0 ± 4.1	
Post-traumatic amnesia, days		22.6 ± 20.6	
Craniotomy or craniectomy, n (%)		1525 (24.0%)	
Acute hospital LOS, days		20.1 ± 16.2	
Inpatient rehabilitation LOS, days		24.8 ± 22.8	
Total LOS (acute + rehabilitation), days		44.5 ± 31.5	

*Values are mean ± standard deviation for continuous variables and numbers with column percentages for categorical variables. Percentages are calculated using the number of participants with non-missing data for each variable as the denominator. Moderate-to-severe traumatic brain injury was defined according to Traumatic Brain Injury Model Systems criteria based on acute Glasgow Coma Scale score in the moderate or severe range, presence of intracranial pathology on neuroimaging, neurosurgical intervention, or prolonged post-traumatic amnesia consistent with at least moderate injury. Mechanisms of injury were grouped into motor vehicle or transport-related causes, falls, violence, and other causes according to the Cause variable in the TBIMS dataset. Age values coded as 777 or 999, education coded as 999, Glasgow Coma Scale values coded as 77, 88, or 999, post-traumatic amnesia coded as 8888 or 9999, and length of stay values coded as 888 or 999 were treated as missing in accordance with the TBIMS data dictionary. Abbreviations: traumatic brain injury (TBI); Traumatic Brain Injury Model Systems (TBIMS); Glasgow Outcome Scale–Extended (GOS-E); Participation Assessment with Recombined Tools–Objective (PART-O); Satisfaction With Life Scale (SWLS); length of stay (LOS).*

**Table 2:** Baseline characteristics by mismatch group at 5 years after TBI. Panel A. Continuous demographic, injury severity, and rehabilitation characteristics at baseline by mismatch group. Panel B. Categorical demographic, pre-injury, injury mechanism, and neurosurgical characteristics at baseline by mismatch group.

Characteristic	Group A (n = 2057)	Group B (n = 495)	Group C (n = 1680)	Group D (n = 1148)	*p*-Value
Age at injury, years	34.6 ± 16.3	43.8 ± 21.5	40.8 ± 16.5	37.0 ± 15.1	<0.001
Education, years	13.7 ± 2.6	12.5 ± 2.7	12.0 ± 2.6	13.2 ± 2.6	<0.001
Initial GCS total	11.2 ± 3.9	11.6 ± 3.9	10.8 ± 4.2	10.6 ± 4.1	<0.001
Post-traumatic amnesia, days	18.0 ± 16.2	19.2 ± 17.3	26.8 ± 24.2	26.0 ± 21.8	<0.001
Acute hospital LOS, days	16.3 ± 11.3	18.3 ± 17.3	23.3 ± 19.9	22.4 ± 16.3	<0.001
Inpatient rehabilitation LOS, days	19.6 ± 14.9	22.1 ± 24.2	29.0 ± 25.2	28.5 ± 26.2	<0.001
Total LOS (acute + rehabilitation), days	35.7 ± 21.4	40.0 ± 32.3	51.8 ± 36.3	50.3 ± 34.1	<0.001
Sex: Female	544 (26.4%)	141 (28.5%)	466 (27.8%)	334 (29.1%)	0.406
Sex: Male	1513 (73.6%)	354 (71.5%)	1213 (72.2%)	813 (70.9%)	0.406
Race: Non-White	468 (22.8%)	165 (33.4%)	590 (35.2%)	311 (27.1%)	<0.001
Race: White	1589 (77.2%)	329 (66.6%)	1088 (64.8%)	837 (72.9%)	<0.001
Mechanism: Fall	405 (19.7%)	163 (33.0%)	406 (24.2%)	221 (19.3%)	<0.001
Mechanism: Motor vehicle/transport	1271 (61.8%)	242 (49.0%)	887 (52.9%)	696 (60.6%)	<0.001
Mechanism: Other	272 (13.2%)	52 (10.5%)	168 (10.0%)	128 (11.1%)	<0.001
Mechanism: Violence	108 (5.3%)	37 (7.5%)	217 (12.9%)	103 (9.0%)	<0.001
Pre-injury employment: Employed	1587 (77.2%)	222 (45.1%)	1017 (60.8%)	893 (77.9%)	<0.001
Pre-injury employment: Homemaker	31 (1.5%)	8 (1.6%)	50 (3.0%)	15 (1.3%)	<0.001
Pre-injury employment: Other	11 (0.5%)	6 (1.2%)	12 (0.7%)	8 (0.7%)	<0.001
Pre-injury employment: Retired	97 (4.7%)	139 (28.3%)	234 (14.0%)	51 (4.4%)	<0.001
Pre-injury employment: Student	234 (11.4%)	43 (8.7%)	88 (5.3%)	75 (6.5%)	<0.001
Pre-injury employment: Unemployed	96 (4.7%)	74 (15.0%)	272 (16.3%)	105 (9.2%)	<0.001
Pre-injury illicit drug use: No	1698 (83.3%)	395 (80.6%)	1215 (73.3%)	907 (79.7%)	<0.001
Pre-injury illicit drug use: Yes	340 (16.7%)	95 (19.4%)	442 (26.7%)	231 (20.3%)	<0.001
Craniotomy or craniectomy: No	1653 (80.4%)	380 (76.8%)	1196 (71.2%)	896 (78.0%)	<0.001
Craniotomy or craniectomy: Yes	404 (19.6%)	115 (23.2%)	484 (28.8%)	252 (22.0%)	<0.001

*Groups: A = high GOS-E, good participation; B = high GOS-E, poor participation; C = low GOS-E, poor participation; D = low GOS-E, good participation. Group sizes: A, n = 2057; B, n = 495; C, n = 1680; D, n = 1148. Values are mean ± standard deviation for continuous variables and numbers with column percentages for categorical variables. Percentages are calculated using the number of participants with non-missing data for each characteristic as the denominator. Groups were defined at 5 years after injury according to global disability on the Glasgow Outcome Scale–Extended (GOS-E) and participation status based on the PART-O total score and productive role engagement. Operational definitions and the hierarchical assignment algorithm are summarized in Supplementary Table S1. The mechanism of injury was categorized as motor vehicle or transport-related, fall, violence, or other, based on the TBIMS Cause variable. Pre-injury employment categories were derived from the TBIMS EMPLOYMENT variable to reflect broad status groups (employed, student, homemaker, unemployed, retired, other). Values coded as non-substantive in the TBIMS data dictionary (for example, 66/88/99, 777/999, and 888/999/8888/9999 depending on the variable) were treated as missing for analysis. *p*-values are from unadjusted global tests comparing the distribution of each characteristic across groups A–D (one-way analysis of variance for continuous variables and chi-square tests for categorical variables). Continuous variables were additionally examined within a 2 × 2 factorial framework (global outcome: high vs. low GOS-E; participation: good vs. poor) using two-way analysis of variance with an interaction term. Tukey-adjusted pairwise comparisons were used when follow-up contrasts were required. Results from the factorial models are reported in Supplementary Table S1. Abbreviations: Traumatic brain injury (TBI); Traumatic Brain Injury Model Systems (TBIMS); Glasgow Outcome Scale–Extended (GOS-E); Participation Assessment with Recombined Tools–Objective (PART-O); Satisfaction With Life Scale (SWLS); Glasgow Coma Scale (GCS); length of stay (LOS).*

**Table 3:** Five-year outcomes by mismatch group. Panel A. Global disability, participation, and employment at 5 years by mismatch group. Panel B. Subjective well-being, mental health, rehospitalization, and social context by mismatch group.

Characteristic	Group A (n = 2057)	Group B (n = 495)	Group C (n = 1680)	Group D (n = 1148)	*p*-Value
GOS-E at 5 years (total score)	7.7 ± 0.5	7.6 ± 0.5	4.7 ± 1.1	5.5 ± 0.8	<0.001
GOS-E at 2 years (total score)	7.2 ± 1.0	6.7 ± 1.4	5.1 ± 1.5	5.8 ± 1.4	<0.001
PART-O total (Rasch score)	60.9 ± 4.2	50.3 ± 7.2	46.2 ± 7.3	59.1 ± 3.8	<0.001
PART-O Productivity domain (Rasch score)	−0.1 ± 0.6	−0.6 ± 0.6	−0.5 ± 0.5	−0.5 ± 0.7	<0.001
PART-O Social Relations domain (Rasch score)	0.5 ± 0.6	0.6 ± 0.6	0.6 ± 0.6	0.6 ± 0.6	<0.001
PART-O Out and About domain (Rasch score)	−0.4 ± 0.5	0.0 ± 0.5	−0.1 ± 0.5	−0.1 ± 0.6	<0.001
Employment at 5 years: Competitively employed	1683 (81.8%)	23 (4.6%)	56 (3.3%)	521 (45.4%)	<0.001
Employment at 5 years: Student	103 (5.0%)	4 (0.8%)	15 (0.9%)	85 (7.4%)	<0.001
Employment at 5 years: Homemaker	47 (2.3%)	11 (2.2%)	29 (1.7%)	41 (3.6%)	<0.001
Employment at 5 years: Retired	206 (10.0%)	205 (41.4%)	970 (57.7%)	454 (39.5%)	<0.001
Employment at 5 years: Unemployed	0 (0.0%)	247 (49.9%)	583 (34.7%)	0 (0.0%)	<0.001
Employment at 5 years: Other/volunteer/special employment	18 (0.9%)	5 (1.0%)	27 (1.6%)	47 (4.1%)	<0.001
Satisfaction With Life Scale (SWLS) total score	27.0 ± 6.2	22.2 ± 8.0	17.2 ± 8.0	22.1 ± 7.6	<0.001
Low life satisfaction (SWLS ≤ 14), n (%)	109 (5.3%)	99 (20.0%)	729 (43.4%)	222 (19.3%)	<0.001
PHQ-9 total score	1.9 ± 3.6	2.8 ± 4.6	7.3 ± 7.3	5.0 ± 6.0	<0.001
PHQ-9 ≥ 10, n (%)	86 (5.6%)	39 (10.4%)	424 (35.1%)	182 (22.6%)	<0.001
GAD-7 total score	2.1 ± 3.9	2.6 ± 4.4	5.7 ± 6.5	4.5 ± 5.7	<0.001
GAD-7 ≥ 10, n (%)	84 (6.9%)	29 (9.7%)	252 (27.3%)	128 (18.9%)	<0.001
Any rehospitalization in past year, n (%)	210 (10.3%)	101 (20.4%)	469 (28.1%)	212 (18.5%)	<0.001
Living situation: Spouse/partner	1051 (51.1%)	119 (24.0%)	511 (30.4%)	543 (47.3%)	<0.001
Living situation: Alone	379 (18.4%)	136 (27.5%)	351 (20.9%)	181 (15.8%)	<0.001
Living situation: Other family	471 (22.9%)	198 (40.0%)	627 (37.3%)	347 (30.2%)	<0.001
Living situation: Someone else	156 (7.6%)	42 (8.5%)	191 (11.4%)	77 (6.7%)	<0.001
Marital status: Single (never married)	899 (43.7%)	226 (45.7%)	690 (41.1%)	461 (40.2%)	<0.001
Marital status: Married	830 (40.4%)	105 (21.2%)	395 (23.5%)	431 (37.5%)	<0.001
Marital status: Divorced	236 (11.5%)	96 (19.4%)	400 (23.8%)	190 (16.6%)	<0.001
Marital status: Separated	40 (1.9%)	18 (3.6%)	102 (6.1%)	41 (3.6%)	<0.001
Marital status: Widowed	48 (2.3%)	50 (10.1%)	93 (5.5%)	24 (2.1%)	<0.001
Marital status: Other	3 (0.1%)	0 (0.0%)	0 (0.0%)	1 (0.1%)	<0.001

*Groups: A = high GOS-E, good participation; B = high GOS-E, poor participation; C = low GOS-E, poor participation; D = low GOS-E, good participation. Values are mean ± standard deviation for continuous variables and numbers with column percentages for categorical variables. Percentages are calculated using the number of participants with non-missing data for each characteristic as the denominator. Group A includes participants with high GOS-E (7–8) and good participation. Group B includes participants with high GOS-E and poor participation. Group C includes participants with low GOS-E (3–6) and poor participation. Group D includes participants with low GOS-E and good participation. Good and poor participation were defined using the PART-O total Rasch score and productive role engagement at 5 years, as described in the Methods and summarized in Supplementary Table S1. Low life satisfaction was defined as an SWLS total score ≤ 14. Clinically significant depressive and anxiety symptoms were defined as PHQ-9 or GAD-7 scores ≥ 10. Rehospitalization refers to any hospital admission in the year preceding the 5-year follow-up. *p*-values are from unadjusted global tests comparing the distribution of each characteristic across groups A–D (one-way analysis of variance for continuous variables and chi-square tests for categorical variables). Continuous variables were additionally examined within a 2 × 2 factorial framework (global outcome: high vs. low GOS-E; participation: good vs. poor) using two-way analysis of variance with an interaction term. Tukey-adjusted pairwise comparisons were used when follow-up contrasts were required. Results from the factorial models are reported in Supplementary Table S1. Abbreviations: Glasgow Outcome Scale–Extended (GOS-E); Participation Assessment with Recombined Tools–Objective (PART-O); Satisfaction With Life Scale (SWLS); Patient Health Questionnaire-9 (PHQ-9); Generalized Anxiety Disorder-7 (GAD-7).*

**Table 4:** Multivariable logistic regression models for mismatch group membership. Panel A. Adjusted odds ratios for membership in mismatch groups B, C, and D compared with Group A. Panel B. Binary logistic regression for poor participation among individuals with good global recovery (Group B vs. Group A).

Predictor	Group B vs. A OR (95% CI)	Group B vs. A *p*-Value	Group C vs. A OR (95% CI)	Group C vs. A *p*-Value	Group D vs. A OR (95% CI)	Group D vs. A *p*-Value
Age at injury (per 10 years)	1.43 (1.26–1.62)	<0.001	1.34 (1.21–1.48)	<0.001	1.16 (1.05–1.28)	0.003
Male sex (vs. female)	0.99 (0.69–1.44)	0.972	0.82 (0.61–1.09)	0.172	0.70 (0.53–0.93)	0.014
Race/ethnicity, non-White (vs. White)	1.88 (1.32–2.68)	<0.001	1.57 (1.18–2.09)	0.002	1.16 (0.87–1.55)	0.312
Education (per year)	0.86 (0.81–0.91)	<0.001	0.85 (0.81–0.90)	<0.001	0.97 (0.92–1.02)	0.189
Pre-injury student (vs. employed)	2.03 (1.05–3.92)	0.034	0.81 (0.46–1.46)	0.489	0.75 (0.44–1.28)	0.288
Pre-injury homemaker (vs. employed)	1.47 (0.51–4.25)	0.482	0.98 (0.41–2.33)	0.956	0.13 (0.03–0.58)	0.008
Pre-injury retired (vs. employed)	5.21 (3.15–8.62)	<0.001	2.42 (1.52–3.83)	<0.001	0.72 (0.41–1.26)	0.252
Pre-injury unemployed (vs. employed)	2.92 (1.62–5.29)	<0.001	2.84 (1.77–4.57)	<0.001	1.27 (0.75–2.13)	0.374
Pre-injury other status (vs. employed)	2.63 (0.84–8.25)	0.097	0.62 (0.19–1.97)	0.413	0.66 (0.20–2.15)	0.492
Pre-injury illicit drug use (yes vs. no)	1.81 (1.15–2.83)	0.010	1.80 (1.26–2.56)	0.001	1.36 (0.95–1.94)	0.096
Admission Glasgow Coma Scale (per point)	0.95 (0.90–1.00)	0.059	1.00 (0.96–1.04)	0.935	0.99 (0.95–1.03)	0.675
Post-traumatic amnesia (per 10 days)	1.11 (0.98–1.25)	0.105	1.12 (1.01–1.23)	0.026	1.12 (1.02–1.23)	0.023
Inpatient rehabilitation LOS (per 10 days)	1.00 (0.88–1.14)	0.969	1.06 (0.96–1.16)	0.254	1.07 (0.97–1.17)	0.166
Neurosurgical procedure (craniotomy/craniectomy)	1.06 (0.71–1.59)	0.771	1.38 (1.00–1.91)	0.047	1.04 (0.75–1.44)	0.806
Fall-related injury (vs. motor vehicle)	1.38 (0.92–2.07)	0.121	1.01 (0.73–1.40)	0.963	0.88 (0.63–1.23)	0.459
Violence-related injury (vs. motor vehicle)	1.46 (0.80–2.64)	0.215	1.16 (0.72–1.85)	0.549	1.26 (0.78–2.04)	0.347
Other mechanism (vs. motor vehicle)	0.83 (0.48–1.45)	0.516	0.66 (0.43–1.00)	0.051	0.90 (0.61–1.32)	0.584
GOS-E at 2 years (per point)	0.70 (0.61–0.80)	<0.001	0.33 (0.30–0.37)	<0.001	0.43 (0.38–0.48)	<0.001
**Predictor**			**Group B vs. A OR (95% CI)**		**Group B vs. A *p*-value**	
Age at injury (per 10 years)			1.38 (1.21–1.58)		<0.001	
Male sex (vs. female)			1.06 (0.71–1.59)		0.761	
Race/ethnicity, non-White (vs. White)			1.82 (1.26–2.62)		0.001	
Education (per year)			0.87 (0.82–0.93)		<0.001	
Pre-injury student (vs. employed)			2.17 (1.11–4.25)		0.023	
Pre-injury homemaker (vs. employed)			1.16 (0.38–3.57)		0.789	
Pre-injury retired (vs. employed)			4.97 (2.95–8.38)		<0.001	
Pre-injury unemployed (vs. employed)			3.14 (1.70–5.77)		<0.001	
Pre-injury other status (vs. employed)			2.47 (0.77–7.88)		0.127	
Pre-injury illicit drug use (yes vs. no)			1.62 (1.01–2.59)		0.047	
Admission Glasgow Coma Scale (per point)			0.94 (0.89–1.00)		0.033	
Post-traumatic amnesia (per 10 days)			1.12 (0.99–1.28)		0.08	
Inpatient rehabilitation LOS (per 10 days)			0.98 (0.86–1.12)		0.739	
Neurosurgical procedure (craniotomy/craniectomy)			1.06 (0.69–1.63)		0.778	
Fall-related injury (vs. motor vehicle)			1.51 (0.98–2.32)		0.059	
Violence-related injury (vs. motor vehicle)			1.82 (0.98–3.37)		0.058	
Other mechanism (vs. motor vehicle)			0.90 (0.51–1.61)		0.728	
GOS-E at 2 years (per point)			0.77 (0.67–0.89)		<0.001	

*Group A = high GOS-E, good participation; Group B = high GOS-E, poor participation; Group C = low GOS-E, poor participation; Group D = low GOS-E, good participation. Panel A reports adjusted odds ratios from a multinomial logistic regression model with mismatch group as the dependent variable and Group A as the reference category. The model was restricted to participants with complete data on all predictors (n = 2257; Group A, n = 884, Group B, n = 233, Group C, n = 681, Group D, n = 459). Panel B reports results from a binary logistic regression contrasting Group B versus Group A among participants with good global recovery, using the same predictor set and complete-case data (n = 1117; Group A, n = 884, Group B, n = 233). Predictors were selected a priori based on clinical relevance and prior literature and included sociodemographic characteristics, pre-injury employment and substance use, acute injury severity indices, rehabilitation length of stay, injury mechanism, and 2-year GOS-E. Odds ratios greater than 1 indicate higher odds of belonging to the specified mismatch group relative to Group A for a one-unit increase in the predictor (or relative to the indicated reference category).*

## Data Availability

No new data were created or analyzed in this study.

## Supplementary Materials

The following supporting information can be downloaded at: https://www.mdpi.com/article/10.3390/medsci14010075/s1. Supplementary Table S1. Operational definitions of mismatch groups and factorial decomposition of continuous outcomes.

## References

[1] Dams-O’Connor K., Juengst S.B., Bogner J., Chiaravalloti N.D., Corrigan J.D., Giacino J.T., Harrison-Felix C.L., Hoffman J.M., Ketchum J.M., Lequerica A.H. (2023). Traumatic brain injury as a chronic disease: Insights from the United States Traumatic Brain Injury Model Systems Research Program. Lancet Neurol..

[2] Corrigan J.D., Hammond F.M., Sander A.M., Kroenke K. (2024). Recognition of Traumatic Brain Injury as a Chronic Condition: A Commentary. J. Neurotrauma.

[3] Ruet A., Bayen E., Jourdan C., Ghout I., Meaude L., Lalanne A., Pradat-Diehl P., Nelson G., Charanton J., Aegerter P. (2019). A Detailed Overview of Long-Term Outcomes in Severe Traumatic Brain Injury Eight Years Post-injury. Front. Neurol..

[4] Mostert C.Q.B., Singh R.D., Gerritsen M., Kompanje E.J.O., Ribbers G.M., Peul W.C., van Dijck J.T.J.M. (2022). Long-term outcome after severe traumatic brain injury: A systematic literature review. Acta Neurochir..

[5] Nelson L.D., Temkin N.R., Barber J., Brett B.L., Okonkwo D.O., McCrea M.A., Giacino J.T., Bodien Y.G., Robertson C., Corrigan J.D. (2023). Functional Recovery, Symptoms, and Quality of Life 1 to 5 Years After Traumatic Brain Injury. JAMA Netw. Open.

[6] Wilson L., Boase K., Nelson L.D., Temkin N.R., Giacino J.T., Markowitz A.J., Maas A., Menon D.K., Teasdale G., Manley G.T. (2021). A Manual for the Glasgow Outcome Scale-Extended Interview. J. Neurotrauma.

[7] Yeatts S.D., Martin R.H., Meurer W., Silbergleit R., Rockswold G.L., Barsan W.G., Korley F.K., Wright D.W., Gajewski B.J. (2020). Sliding Scoring of the Glasgow Outcome Scale-Extended as Primary Outcome in Traumatic Brain Injury Trials. J. Neurotrauma.

[8] McMillan T., Wilson L., Ponsford J., Levin H., Teasdale G., Bond M. (2016). The Glasgow Outcome Scale—40 years of application and refinement. Nat. Rev. Neurol..

[9] Whiteneck G.G., Dijkers M.P., Heinemann A.W., Bogner J.A., Bushnik T., Cicerone K.D., Corrigan J.D., Hart T., Malec J.F., Millis S.R. (2011). Development of the Participation Assessment with Recombined Tools–Objective for use after traumatic brain injury. Arch. Phys. Med. Rehabil..

[10] Huebner R.A., Johnson K., Bennett C.M., Schneck C. (2003). Community participation and quality of life outcomes after adult traumatic brain injury. Am. J. Occup. Ther..

[11] Erler K.S., Whiteneck G.G., Juengst S.B., Locascio J.J., Bogner J.A., Kaminski J., Giacino J.T. (2018). Predicting the Trajectory of Participation After Traumatic Brain Injury: A Longitudinal Analysis. J. Head Trauma Rehabil..

[12] Kalpinski R.J., Williamson M.L.C., Elliott T.R., Berry J.W., Underhill A.T., Fine P.R. (2013). Modeling the prospective relationships of impairment, injury severity, and participation to quality of life following traumatic brain injury. BioMed Res. Int..

[13] Andelic N., Howe E.I., Hellstrøm T., Sanchez M.F., Lu J., Løvstad M., Røe C. (2018). Disability and quality of life 20 years after traumatic brain injury. Brain Behav..

[14] Retel Helmrich I.R.A., van Klaveren D., Andelic N., Lingsma H., Maas A., Menon D., Polinder S., Røe C., Steyerberg E.W., Van Veen E. (2022). Discrepancy between disability and reported well-being after traumatic brain injury. J. Neurol. Neurosurg. Psychiatry.

[15] Ritchie L., Wright-St Clair V.A., Keogh J., Gray M. (2014). Community integration after traumatic brain injury: A systematic review of the clinical implications of measurement and service provision for older adults. Arch. Phys. Med. Rehabil..

[16] Malone C., Erler K.S., Giacino J.T., Hammond F.M., Juengst S.B., Locascio J.J., Nakase-Richardson R., Verduzco-Gutierrez M., Whyte J., Zasler N. (2019). Participation Following Inpatient Rehabilitation for Traumatic Disorders of Consciousness: A TBI Model Systems Study. Front. Neurol..

[17] Hauger S.L., Borgen I.M.H., Forslund M.V., Kleffelgård I., Andelic N., Løvstad M., Perrin P.B., Røe C., Fure S.C.R. (2023). Participation in the Chronic Phase after Traumatic Brain Injury: Variations and Key Predictors. J. Clin. Med..

[18] Venkatesan U.M., Adams L.M., Rabinowitz A.R., Agtarap S., Bombardier C.H., Bushnik T., Chiaravalloti N.D., Juengst S.B., Katta-Charles S., Perrin P.B. (2023). Societal Participation of People with Traumatic Brain Injury Before and During the COVID-19 Pandemic: A NIDILRR Traumatic Brain Injury Model Systems Study. Arch. Phys. Med. Rehabil..

[19] Diener E., Emmons R.A., Larsen R.J., Griffin S. (1985). The Satisfaction with Life Scale. J. Personal. Assess..

[20] Corrigan J.D., Bogner J.A., Mysiw W.J., Clinchot D., Fugate L. (2001). Life satisfaction after traumatic brain injury. J. Head Trauma Rehabil..

[21] Kreitzer N.P., Hart K., Lindsell C.J., Manley G.T., Dikmen S.S., Ratcliff J.J., Yue J.K., Adeoye O.M. (2019). A Comparison of Satisfaction with Life and the Glasgow Outcome Scale-Extended After Traumatic Brain Injury: An Analysis of the TRACK-TBI Pilot Study. J. Head Trauma Rehabil..

[22] Dijkers M.P., Harrison-Felix C., Marwitz J.H. (2010). The traumatic brain injury model systems: History and contributions to clinical service and research. J. Head Trauma Rehabil..

[23] Hammond F.M., Malec J.F. (2010). The Traumatic Brain Injury Model Systems: A longitudinal database, research, collaboration and knowledge translation. Eur. J. Phys. Rehabil. Med..

[24] Corrigan J.D., Cuthbert J.P., Whiteneck G.G., Dijkers M.P., Coronado V., Heinemann A.W., Harrison-Felix C., Graham J.E. (2012). Representativeness of the Traumatic Brain Injury Model Systems National Database. J. Head Trauma Rehabil..

[25] Cuthbert J.P., Corrigan J.D., Whiteneck G.G., Harrison-Felix C., Graham J.E., Bell J.M., Coronado V.G. (2012). Extension of the representativeness of the Traumatic Brain Injury Model Systems National Database: 2001 to 2010. J. Head Trauma Rehabil..

[26] Pretz C.R., Dams-O’Connor K. (2013). Longitudinal description of the glasgow outcome scale-extended for individuals in the traumatic brain injury model systems national database: A National Institute on Disability and Rehabilitation Research traumatic brain injury model systems study. Arch. Phys. Med. Rehabil..

[27] Tso S., Saha A., Cusimano M.D. (2021). The Traumatic Brain Injury Model Systems National Database: A Review of Published Research. Neurotrauma Rep..

[28] Corrigan J.D., Selassie A.W., Lineberry L.A., Millis S.R., Wood K.D., Pickelsimer E.E., Rosenthal M. (2007). Comparison of the Traumatic Brain Injury (TBI) Model Systems national dataset to a population-based cohort of TBI hospitalizations. Arch. Phys. Med. Rehabil..

[29] von Elm E., Altman D.G., Egger M., Pocock S.J., Gøtzsche P.C., Vandenbroucke J.P., STROBE Initiative (2007). The Strengthening the Reporting of Observational Studies in Epidemiology (STROBE) statement: Guidelines for reporting observational studies. Lancet.

[30] Benchimol E.I., Smeeth L., Guttmann A., Harron K., Moher D., Petersen I., Sørensen H.T., von Elm E., Langan S.M., RECORD Working Committee (2015). The REporting of studies Conducted using Observational Routinely-collected health Data (RECORD) statement. PLoS Med..

[31] Bogner J., Bellon K., Kolakowsky-Hayner S.A., Whiteneck G. (2013). Participation assessment with recombined tools-objective (PART-O). J. Head Trauma Rehabil..

[32] Milo R.B., Martinez N., Asmus T., Lee J., Soon K., Milo J., Calero P. (2025). Translated versions of the English satisfaction with life scale (SWLS) among adult participants: A systematic review. BMC Psychol..

[33] Chan B.K.C. (2018). Data analysis using R programming. Biostatistics for Human Genetic Epidemiology; Advances in Experimental Medicine and Biology.

[34] World Medical Association (2013). World Medical Association Declaration of Helsinki: Ethical principles for medical research involving human subjects. JAMA.

[35] Keyser-Marcus L.A., Bricout J.C., Wehman P., Campbell L.R., Cifu D.X., Englander J., High W., Zafonte R.D. (2002). Acute predictors of return to employment after traumatic brain injury: A longitudinal follow-up. Arch. Phys. Med. Rehabil..

[36] Howe E.I., Andelic N., Perrin P.B., Røe C., Sigurdardottir S., Arango-Lasprilla J.C., Lu J., Løvstad M., Forslund M.V. (2018). Employment Probability Trajectories Up To 10 Years After Moderate-To-Severe Traumatic Brain Injury. Front. Neurol..

[37] Fure S.C.R., Howe E.I., Andelic N., Brunborg C., Sveen U., Røe C., Rike P.O., Olsen A., Spjelkavik Ø., Ugelstad H. (2021). Cognitive and vocational rehabilitation after mild-to-moderate traumatic brain injury: A randomised controlled trial. Ann. Phys. Rehabil. Med..

[38] Simpson G.K., McRae P., Gates T.M., Daher M., Johnston D., Cameron I.D. (2023). A vocational intervention that enhances return to work after severe acquired brain injury: A pragmatic trial. Ann. Phys. Rehabil. Med..

[39] Ponsford J., Downing M.G., O’Kearney E., Bedekar Y., Hilton G., Mortimer D., Fossey E., Barclay L., Olver J., Castle W. (2025). Early intervention vocational rehabilitation for return to work following traumatic injury: A randomized controlled trial. Ann. Phys. Rehabil. Med..

[40] Juengst S.B., Kumar R.G., Venkatesan U.M., O’Neil-Pirozzi T.M., Evans E., Sander A.M., Klyce D., Agtarap S., Erler K.S., Rabinowitz A.R. (2024). Predictors of Multidimensional Profiles of Participation After Traumatic Brain Injury: A TBI Model Systems Study. J. Head Trauma Rehabil..

[41] Guerrette M.C., McKerral M. (2023). Predictors of Social Participation Outcome after Traumatic Brain Injury Differ According to Rehabilitation Pathways. J. Neurotrauma.

[42] Lu J., Rasmussen M.S., Sigurdardottir S., Forslund M.V., Howe E.I., Fure S.C.R., Løvstad M., Overeem R., Røe C., Andelic N. (2023). Community Integration and Associated Factors 10 Years after Moderate-to-Severe Traumatic Brain Injury. J. Clin. Med..

[43] Moreno A., Sun H., Mckerral M. (2023). Social participation and health-related quality of life before and during the second wave of the Covid-19 pandemic in individuals with traumatic brain injury: A follow-up exploratory correlational study. J. Rehabil. Med..

[44] Walker W.C., Ketchum J.M., Marwitz J.H., Chen T., Hammond F., Sherer M., Meythaler J. (2010). A multicentre study on the clinical utility of post-traumatic amnesia duration in predicting global outcome after moderate-severe traumatic brain injury. J. Neurol. Neurosurg. Psychiatry.

[45] Wise E.K., Mathews-Dalton C., Dikmen S., Temkin N., Machamer J., Bell K., Powell J.M. (2010). Impact of traumatic brain injury on participation in leisure activities. Arch. Phys. Med. Rehabil..

[46] Kersey J., Terhorst L., Wu C.Y., Skidmore E. (2019). A Scoping Review of Predictors of Community Integration Following Traumatic Brain Injury: A Search for Meaningful Associations. J. Head Trauma Rehabil..

[47] Sander A.M., Lequerica A.H., Ketchum J.M., Hammond F.M., Gary K.W., Pappadis M.R., Felix E.R., Johnson-Greene D., Bushnik T. (2018). Race/Ethnicity and Retention in Traumatic Brain Injury Outcomes Research: A Traumatic Brain Injury Model Systems National Database Study. J. Head Trauma Rehabil..

[48] Wroblewski T.H., Ononogbu-Uche F.C., Jagtiani P., Calixte R.M.E., Roberts M.C., Barr P.B., Bigdeli T.B., Barthélemy E.J. (2025). Inequities in Neuropsychiatric Outcomes After Brain Trauma in the All of Us Database. JAMA Netw. Open.

[49] Cicerone K.D. (2004). Participation as an outcome of traumatic brain injury rehabilitation. J. Head Trauma Rehabil..

