# Supplementary material for: Good Recovery, Poor Participation? A Secondary Analysis of the Dissociation Between Global Disability and Real-Life Participation Five Years After Traumatic Brain Injury
Source: Med Sci (Basel). 2026 Feb 9;14(1):75. doi: 10.3390/medsci14010075 (PMC12921937; doi:10.3390/medsci14010075)
Supplement: Supplementary file 1 [file medsci-14-00075-s001.zip › medsci-4130233-supplementary.pdf]

Supplementary Table S1. Operational definitions of mismatch groups and factorial decomposition of continuous outcomes. P-values in Panel B are from two-way analysis of variance models with main effects of global outcome (high vs low GOS-E) and participation (good vs poor) and their interaction, using Type III sums of squares. N varies because models use available-case data for each variable. Abbreviations: GOS-E, Glasgow Outcome Scale–Extended; PART-O, Participation Assessment with Recombined Tools–Objective; SWLS, Satisfaction With Life Scale; PHQ-9, Patient Health Questionnaire-9; GAD-7, Generalized Anxiety Disorder-7; GCS, Glasgow Coma Scale; LOS, length of stay.

Panel A. Operational definitions and hierarchical assignment algorithm (5-year outcomes).

Component	Operational definition
PART-O total Rasch 25th percentile (analytic cohort, N = 6,363)	49.00
PART-O total Rasch median (analytic cohort, N = 6,363)	55.28
High global outcome	GOS-E at 5 years equals 7 to 8.
Low global outcome	GOS-E at 5 years equals 3 to 6.
Poor participation (assigned first)	EMPLOYMENTF equals 10 (unemployed) or PART-O total Rasch score is at or below 49.00.
Good participation (among remaining)	PART-O total Rasch score is at or above 55.28.
Intermediate participation (remaining)	PART-O total Rasch score is between 49.00 and 55.28 and the participant is not unemployed.
Group A	High global outcome and good participation.
Group B	High global outcome and poor participation.
Group C	Low global outcome and poor participation.
Group D	Low global outcome and good participation.

Panel B. Two-way analysis of variance for continuous variables (global outcome × participation).

Variable	Global outcome p	Participation p	Interaction p	N used
Age at injury, years	0.525	<0.001	<0.001	5362
Education, years	<0.001	<0.001	0.805	5357
Initial GCS total	<0.001	0.053	0.529	2883
Post-traumatic amnesia, days	<0.001	0.148	0.852	4650
Acute hospital LOS, days	<0.001	0.004	0.290	5377
Inpatient rehabilitation LOS, days	<0.001	0.014	0.224	5379
Total LOS (acute plus rehabilitation), days	<0.001	0.004	0.149	5297
GOS-E at 5 years (total score)	<0.001	<0.001	<0.001	5380
GOS-E at 2 years (total score)	<0.001	<0.001	0.033	4942
PART-O total (Rasch score)	<0.001	<0.001	<0.001	5380
PART-O Productivity domain (Rasch score)	<0.001	<0.001	<0.001	5380
PART-O Social Relations domain (Rasch score)	<0.001	0.031	0.018	5380
PART-O Out and About domain (Rasch score)	<0.001	<0.001	<0.001	5380
SWLS total score	<0.001	<0.001	0.596	5380
PHQ-9 total score	<0.001	<0.001	0.001	3923
GAD-7 total score	<0.001	<0.001	0.151	3116
